# Discrimination of Ultrasonic Vocalizations by CBA/CaJ Mice (*Mus musculus*) Is Related to Spectrotemporal Dissimilarity of Vocalizations

**DOI:** 10.1371/journal.pone.0085405

**Published:** 2014-01-09

**Authors:** Erikson G. Neilans, David P. Holfoth, Kelly E. Radziwon, Christine V. Portfors, Micheal L. Dent

**Affiliations:** 1 Department of Psychology, University at Buffalo, the State University of New York, Buffalo, New York, United States of America; 2 School of Biological Sciences, Washington State University-Vancouver, Vancouver, Washington, United States of America; University of South Florida, United States of America

## Abstract

The function of ultrasonic vocalizations (USVs) produced by mice (*Mus musculus*) is a topic of broad interest to many researchers. These USVs differ widely in spectrotemporal characteristics, suggesting different categories of vocalizations, although this has never been behaviorally demonstrated. Although electrophysiological studies indicate that neurons can discriminate among vocalizations at the level of the auditory midbrain, perceptual acuity for vocalizations has yet to be determined. Here, we trained CBA/CaJ mice using operant conditioning to discriminate between different vocalizations and between a spectrotemporally modified vocalization and its original version. Mice were able to discriminate between vocalization types and between manipulated vocalizations, with performance negatively correlating with spectrotemporal similarity. That is, discrimination performance was higher for dissimilar vocalizations and much lower for similar vocalizations. The behavioral data match previous neurophysiological results in the inferior colliculus (IC), using the same stimuli. These findings suggest that the different vocalizations *could* carry different meanings for the mice. Furthermore, the finding that behavioral discrimination matched neural discrimination in the IC suggests that the IC plays an important role in the perceptual discrimination of vocalizations.

## Introduction

Many animals, including humans, use sound to communicate, and the vocal repertoire of a species is often quite large (e.g. [1]). This suggests that different vocalizations convey different information to receivers. The vervet monkey (*Chlorocebus pygerythrus*) is a classic example of this; scouts emit one vocalization for a flying predator and another for a terrestrial predator [2]. Moreover, the finding that vervet monkeys have specific behaviors for each vocalization indicates that they can discriminate between the vocalizations and interpret their meaning. In many other animal species, however, the meaning of different vocalizations is not known and it is not clear whether different vocalizations can be discriminated.

Mice (*Mus musculus*) produce an array of ultrasonic vocalizations (USVs) in a variety of behavioral contexts. Males emit USVs in the presence of females, females produce them in the presence of other females, and infants produce them when separated from their mothers (reviewed in [3]). The vocalizations emitted by mice have been classified into syllable types based on spectrotemporal characteristics, but the numbers and types of categories vary widely [3], [4], [5], [6], [7]. Moreover, these categories have been determined by visual or statistical analyses of spectrograms rather than assessing whether mice can discriminate between the different syllables. The discrimination ability of mice for ultrasonic vocalizations is not yet known.

Playback studies have been useful for establishing the functional importance of mouse USVs, but small effect sizes, habituation, and difficulty interpreting results all hinder their usefulness in understanding mouse communication. Psychophysical experiments, on the other hand, allow us to test the perceptual limits of discriminating between acoustic stimuli [8]. Here, we used psychophysical methods to test the ability of mice to discriminate USVs.

We used the same stimuli that were used to examine neural selectivity to vocalizations in the inferior colliculus (IC) of mice [9]. Holmstrom and colleagues presented awake and restrained mice with four different vocalizations and spectrotemporal manipulations of those vocalizations. These USVs elicited heterogeneous patterns of responses across the neural population and within individual neurons. Neurons also responded differently to unaltered calls compared to those that had modified spectrotemporal properties, suggesting a distinct neural representation of each vocalization. These results suggested that the IC is critical for the encoding of behaviorally relevant sounds, even though the behavioral relevancy of those sounds has not yet been determined. As a first attempt to understand how trained, reliable mouse observers perceive ultrasonic calls, we used psychoacoustic techniques to test the discrimination ability for USVs. We found that the mice were able to discriminate between vocalizations with performance that was always better than chance and that performance was best for USVs that were spectrotemporally dissimilar. These behavioral results are similar to the neural results obtained in the IC, using the same stimuli, which suggests that the IC plays an important role in the perceptual discrimination of vocalizations.

## Materials and Methods

### Ethics Statement

All procedures were approved by the University at Buffalo, SUNY’s Institutional Animal Care and Use Committee under protocol PSY13056N.

### Subjects

We tested how well five mice with normal hearing abilities (CBA/CaJ strain; 3 males, 2 females) could discriminate different mouse USVs. Training began when the mice were approximately 6 months old and the experiments lasted approximately 12 months. The mice were housed separately and kept on a reversed day/night cycle (lights off at 6 am and on at 6 pm). The mice were tested during the dark portion of their cycle. They were water restricted and kept at approximately 85% of their free-drinking weight during the course of the experiment. The animals had unrestricted access to food, except while they were participating in the experiments. The mice were bred at the University at Buffalo, SUNY and all procedures were approved by the University at Buffalo, SUNY’s Institutional Animal Care and use Committee.

### Apparatus

The mice were tested in a wire cage (23×39×15.5 cm) placed in a sound-attenuated chamber (53.5×54.5×57 cm) lined with 4-cm thick Sonex sound attenuating foam (Illbruck Inc., Minneapolis, MN). The chamber was illuminated at all times by a small lamp with a 25-W white light bulb and the behavior of the animals during test sessions was monitored by an overhead web camera (Logitech QuickCam Pro, Model 4000). The test cage consisted of an electrostatic speaker (Tucker-Davis Technologies (TDT), Gainesville, FL, Model ES1), a response dipper (Med Associated Model ENV-302M-UP), and two nose poke holes surrounded by infrared sensors (Med Associates Model ENV-254).

The experiments were controlled by Dell Dimension 3100 computers operating TDT modules and software. Stimuli were sent through an RP2 signal processor, an SA1 power amplifier, a PA5 programmable attenuator, and finally to the speaker. Inputs to and outputs from the testing cages were controlled via RP2 and RX6 processors. Power supplies were used to drive the dipper (Elenco Precision, Wheeling, IL, Model XP-603) and infrared sensors (Elenco Precision, Model XP-650). Custom MATLAB and TDT RPvds software programs were used to control the hardware.

### Stimuli

**Figure 1:**
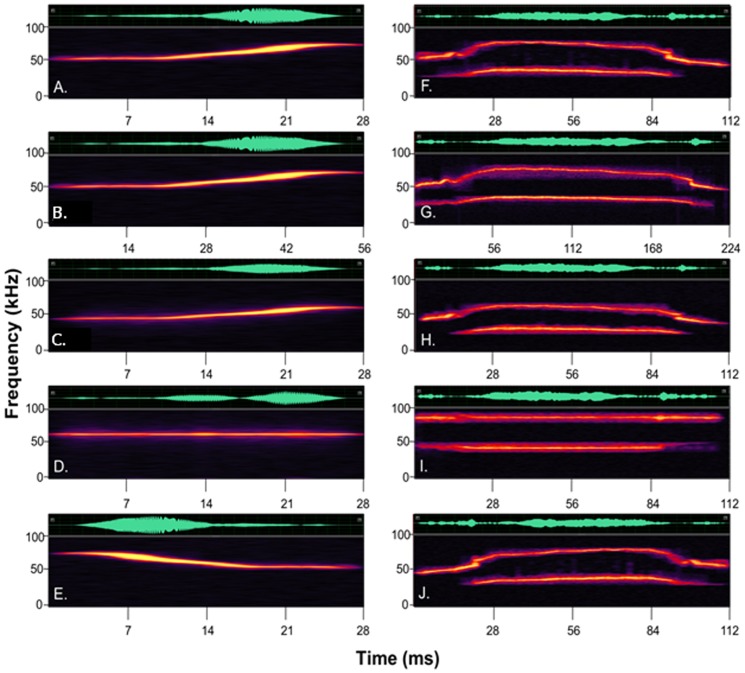
Vocalization spectrograms. Spectrograms of two unmodified vocalizations (A = Male Upsweep and F = 40 kHz Harm/2 Jump), and those same two vocalizations doubled in duration (B and G), lowered in frequency by 20% (C and H), with frequency modulation removed (D and I), and reversed in time (E and J).

We used the same vocalizations used by [9], plus one additional vocalization not included in that study but acquired in their laboratory. Using the same stimuli allows us to more closely compare the neural correlates of call discrimination in mice and the behavioral data collected from this experiment. The vocalizations were recorded from CBA/CaJ mice during social interactions and analyzed with custom-written MATLAB code implementing a harmonic state-space signal model and the extended Kalman smoother [10]. The stimuli were synthesized in this way to reduce background noise and allow for manipulation of individual parameters in each of the vocalizations. We used five vocalization types, named based on the presence/absence of harmonics and jumps in frequency (all produced by males), or simpler sweep shapes produced by a male or a female: F Upsweep, M Upsweep, 30 kHz Harm/0 Jump, 30 kHz Harm/1 Jump, and 40 kHz Harm/2 Jump. Each of these five vocalizations was also manipulated in eight ways: the fundamental frequency was raised by 10 and 20% and lowered by 10 and 20%, the frequency modulation was removed, the entire vocalization was reversed, and the vocalizations were doubled and halved in duration (while maintaining the original frequency). We manipulated these acoustic parameters because they known to be important cues in tasks such as auditory scene segregation (e.g. [11]; [12]). Stimuli were presented at approximately 65 dB SPL, measured at the position where the mouse’s head would normally be during testing. Two of the original vocalizations with several of these manipulations are shown in Figure 1. Sound pressure levels were calculated using an ultrasound recording system (Avisoft Model USG116-200) and Raven Pro (v 1.3, Cornell University) software.

### Procedures

Mice were trained using a go-no-go operant conditioning procedure on a discrimination task. Subjects listened to one vocalization presented repeatedly and were required to indicate when they heard any other stimulus type. The first stage in the training process was to shape the mice to nose poke to the observation hole and then approach the dipper for the chocolate Ensure/water reinforcer. The animals were then trained to repeatedly poke to the observation hole until they heard a vocalization, after which they would nose poke to the report hole for the reinforcement. Next, catch trials were phased into the training and the waiting interval was systematically increased. Finally, a repeating background vocalization (different from the target vocalization) was phased into the training in small intensity increments from session to session.

During testing, the mouse began a trial by nose poking through the observation nose-poke hole two times, which initiated a variable waiting interval ranging from 1 to 4 s. During this time, a repeating background of one vocalization alternating with silence was presented repeatedly at a rate of once every 200 ms. After the waiting interval, a single test stimulus was presented, alternating with the background stimulus vocalization two times. In the “go” condition, the target stimulus was either a different vocalization or a manipulated version of the same vocalization. If the mouse discriminated this change between background and target, it was required to nose poke through the report nose-poke hole within 2 s of the onset of the target. In this trial type, a “hit” was recorded if the mouse correctly responded within the response window and the animal received 0.01 ml of Ensure or water as a reinforcement. A “miss” was recorded if the mouse failed to nose poke through the report hole within 2 s. If the mouse responded to the report nose-poke hole during the waiting interval, the trial was aborted and the mouse received a 3-5-s timeout, during which no stimuli would play.

Approximately 30% of all trials were “no go”, or catch trials. Here, the repeating background continued to be presented during the response phase. These trials were required to measure the false alarm rate and calculate the animal’s response bias. If the subject nose poked to the report hole during a catch trial, a “false alarm” was recorded and the mouse was punished with a 3-s timeout interval. However, if the subject continued to nose poke to the observation hole, a “correct rejection” was recorded and the next trial would begin immediately. In either case, no reinforcement was given. Chance performance was represented by the animal’s false alarm rate. Sessions were excluded from analysis if the percentage of false alarms was greater than 20%. Using the criterion of at least 80% hit rate on target trials and below 20% false alarm rate assures that the mice are under stimulus control [8]. Approximately 30% of all sessions were thrown out due to high false alarm rate. There are two reasons to not include these data: First, including these sessions would lead to a false impression of the discrimination acuity found in this strain of mouse. Second, when the false alarm rates are above 20%, it is difficult to determine whether the animals are responding to the stimuli or just randomly nose-poking due to some motivational or attentional bias.

The mice were tested on two 30-min sessions/day, five to six days per week. Typically, the mice ran between 50 and 100 trials per session. All mice were tested on all stimuli in a random order, and a different random order was used for each subject. Testing on each vocalization background continued until results from at least 20 trials of each target type comparison were collected. Those results were used to calculate the percent correct discrimination performance for each vocalization versus every other vocalization and versus every vocalization manipulation.

### Data Analysis

Signal detection analysis was performed to factor out the animals’ motivational biases. At least 40 trials were obtained for each stimulus comparison for each mouse, where one stimulus was the background and the other was the target for 20 trials, and the reverse was true for another 20 trials. Mean hit rates were calculated for each animal on each stimulus comparison, and false alarms were tracked from session to session to ensure that the animals remained reliable observers under stimulus control. Repeated-measures ANOVAs were used to compare performance across all stimulus types, and Holm-Sidak post-hoc analyses were conducted for pairwise comparisons.

Once discrimination performance for all vocalization combinations (each of the five vocalizations against every other vocalization) was complete, a multidimensional analysis (Proxscal) was conducted. Each cell in a discrimination performance matrix for each mouse contained the responses from a single session involving the discrimination percentage from the first 20 presentations of a single pair of USVs. Once testing was complete, similar to the techniques used by [13], a single matrix representing the mean results across the five different USVs was calculated. The values in the diagonal (i.e., performances from “same” trials) were discarded. Asymmetrical discrimination responses were found across all mice and USV conditions. That is, the discrimination responses varied when a call was the background or target in the USV pairing, although no systematic explanation for this could be found, and asymmetries differed in direction across animals. This required using a full-matrix analysis of discrimination performances, which can be done using Proxscal in SPSS. From this behaviorally derived matrix, the Euclidean distances between the vocalizations were calculated.

Spectrotemporal similarities between the vocalization types and between the vocalizations and their manipulated versions were measured using the cross correlation tool from RAVEN software (Cornell Lab of Ornithology, Ithaca, NY, v. 1.3, biased setting). Those spectrotemporal similarity calculations, along with the behavioral discrimination performance measures, were used for a nonlinear regression analysis.

## Results

**Figure 2:**
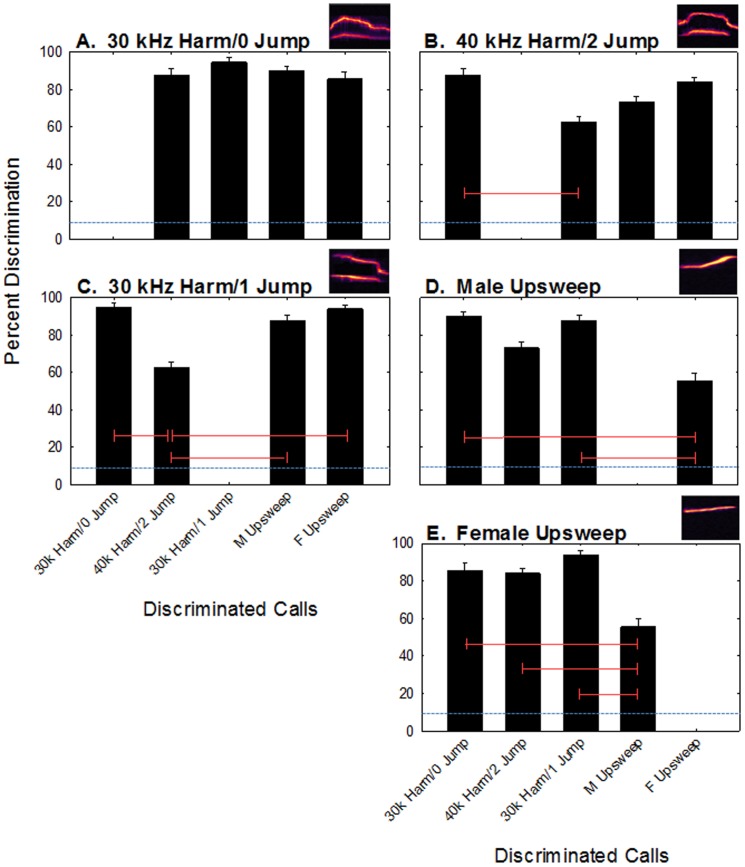
Discrimination of vocalizations. Mean discrimination performance across subjects for the five vocalizations types (A-E) against all other vocalizations. The blue horizontal dashed lines represent chance level performance. Error bars are between-subject standard errors. The red horizontal lines connect significantly different bars. The missing bar in each of the graphs is when the stimulus was used as the background.

We found that the mice generally were able to discriminate between vocalizations (Figure 2). Mean discrimination performance ranged from 56–95% across all vocalizations, while the false alarm rate averaged 9.8% (SE  =  0.1%) across all animals and sessions. A one-way repeated measures ANOVA revealed significant differences in discrimination abilities across the five stimuli (*F*_9,36_  =  8.82, *p* < 0.001). A Holm-Sidak test revealed several significant pair-wise comparisons (Figure 2). Generally, the two upsweep vocalizations were difficult to discriminate from one another (*p* > 0.05) but were easily discriminated from the harmonic vocalizations (*p* < 0.05), while the harmonic vocalizations were easily discriminated from the upsweeps (*p* < 0.05), but the mice had some difficulty discriminating among the different harmonic vocalizations (*p* > 0.05). These discrimination performances do not appear to be due to an experience-dependent confound. The mice took an average of 5 sessions (∼200 trials) to reach criterion and this number did not systematically change as the experiment continued. Specifically, discrimination performance during training and testing were nearly identical for all mice at the beginning of the experiment and at the end of the experiment.

**Figure 3:**
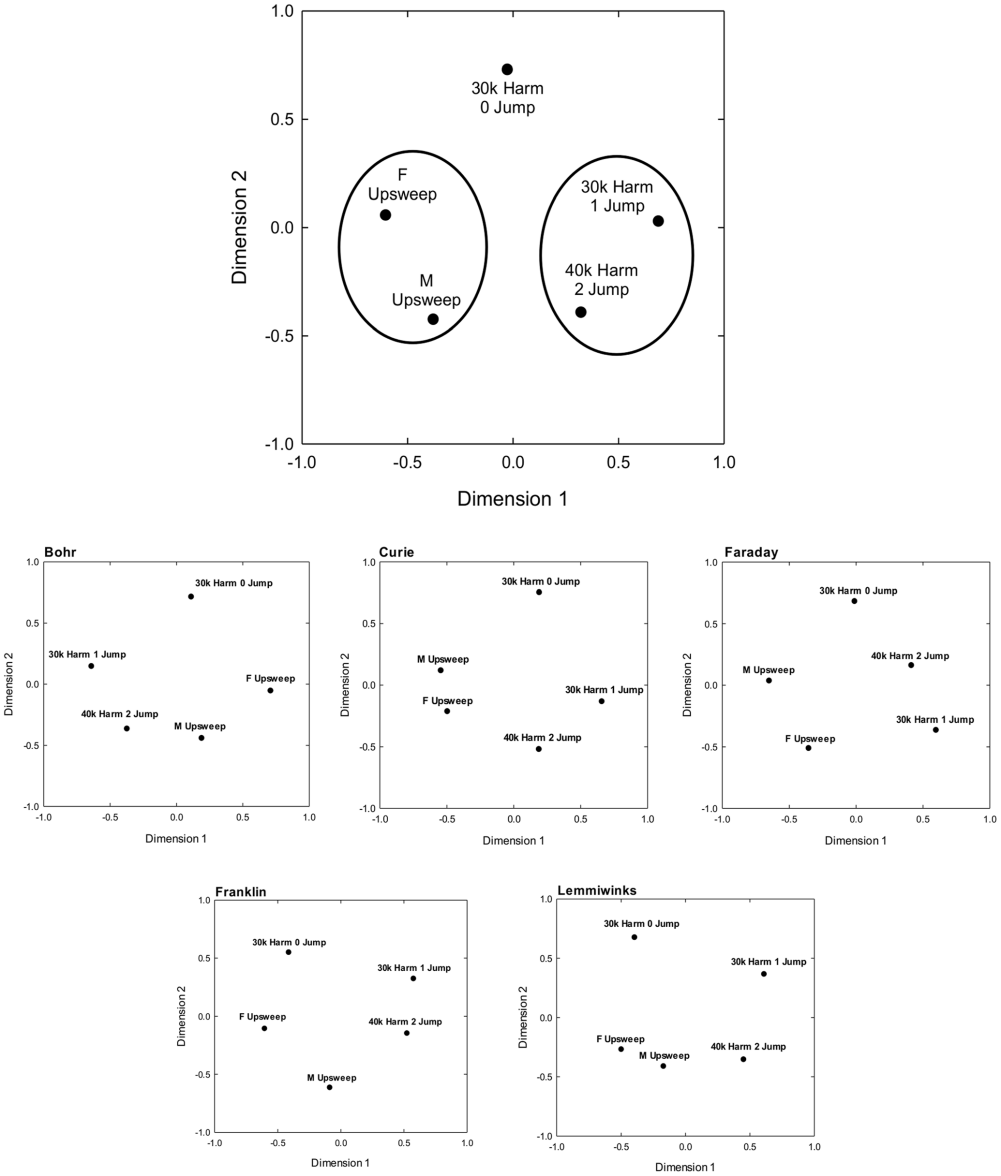
Multidimensional analysis of vocalization discrimination. At the top, multidimensional map of mean vocalization discrimination obtained from matrices of discrimination performance for the five vocalization types. Circles connect vocalizations with the two smallest Euclidian distances. At the bottom are the five individual perceptual maps generated for each mouse across the five vocalization types.

The mean percent correct discrimination performance matrices for every vocalization combination were used to calculate a multidimensional map of vocalization perception in the mice (Figure 3). A two-dimensional map of similarity was conducted from this analysis, accounting for 78% of the dispersion, with the first and second dimensions accounting for 45% and 33% respectively. Along dimension 1 (accounting for slightly more variance), the USVs appear to be divided along the presence of transient frequency “jumps” in the vocalization. Vocalizations with no harmonics or jumps were in the negative portion of dimension 1, while the vocalizations with harmonics and jumps were placed in the positive portion of dimension 1. The one harmonic vocalization with no jumps was in-between the other two sets of vocalizations. It is less clear which acoustic cues the mice are using along the second dimension. It would appear that along dimension 2, the USVs are separated by the spectrotemporal differences found between the start and end of the call. The positive portion of the dimension contains the only USV with the same beginning and end frequency (30k Harm, 0 Jump; Δ  =  0 Hz). The calls in the middle of the second dimension (F Upsweep and 30k Harm, 1 Jump) are found to have ∼5–10 kHz of frequency separation, whereas the calls in the negative portion of the dimension (M Upsweep and 40k Harm, 2 Jump) were found to have the largest frequency separation, about a ∼15–20 kHz change in frequency. These results support the hypotheses stated from [9], that the auditory system may process USVs by the distortion products generated from the beginning and end frequencies of the call. Vocalizations separated by the two smallest Euclidian distances are circled. Based on behavioral discrimination performance alone, the pairs of M Upsweep and F Upsweep and 30kHarm/1Jump and 40kHarm/2Jump were deemed as more similar to each other than the other vocalization comparisons.

**Figure 4:**
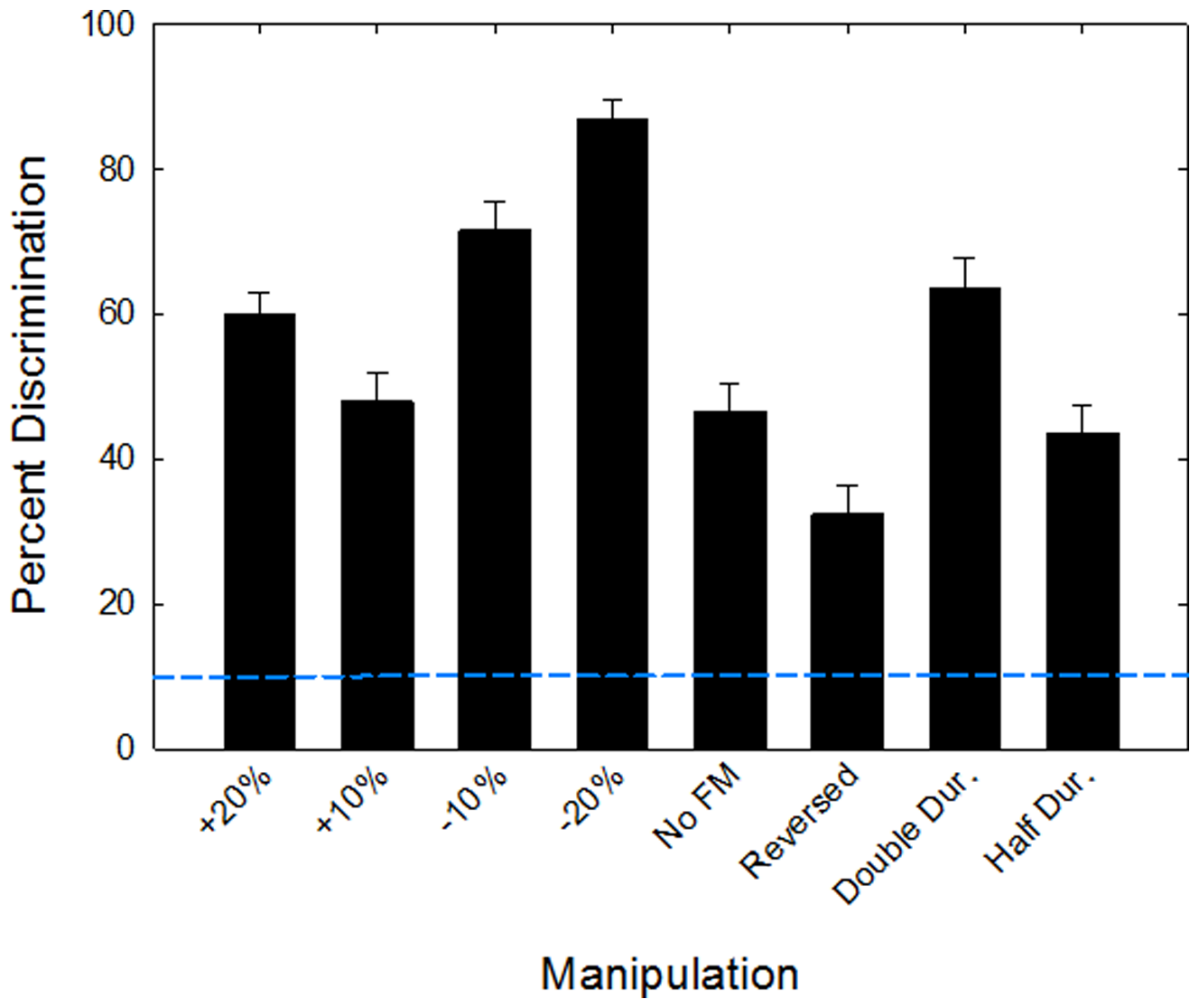
Discrimination of manipulated vocalizations. Mean discrimination performance across subjects and across the five background vocalization types for the eight vocalization manipulations. Error bars are between-subjects standard errors. The blue horizontal dashed lines represent chance level performance.

Discrimination performance for the manipulated vocalizations also varied across conditions (Figure 4), with percent correct ranging from 32–86%, averaged across the five vocalization types. There was a significant difference between the five background vocalization types (*F*_4,28_  =  36.57, *p*<0.001), the eight vocalization manipulations (*F*_7,28_  =  26.04, *p*<0.001), and a significant interaction between the two variables (*F*_28,112_  =  8.16, *p*<0.001). In general, discrimination between the unaltered and manipulated vocalizations was most difficult for the upsweeps (*p* > 0.05) and least difficult for the harmonic vocalizations (*p* < 0.05). The manipulations that were most difficult to discriminate from the unaltered vocalizations were reversing and compressing the vocalizations, followed by removing the frequency modulation and increasing the frequency by 10%. The easiest manipulations to discriminate from the unaltered vocalizations were lowering the frequency of the vocalizations by 20%, lowering the frequency by 10%, doubling the duration, and increasing the frequency by 20%. Regardless of the manipulation, all discriminations were well above chance performance (false alarm rate  =  9.8%). Furthermore, discrimination performance was quite consistent across mice, indicated by the relatively low standard error values across conditions (SE_mean_  =  4.55).

**Figure 5:**
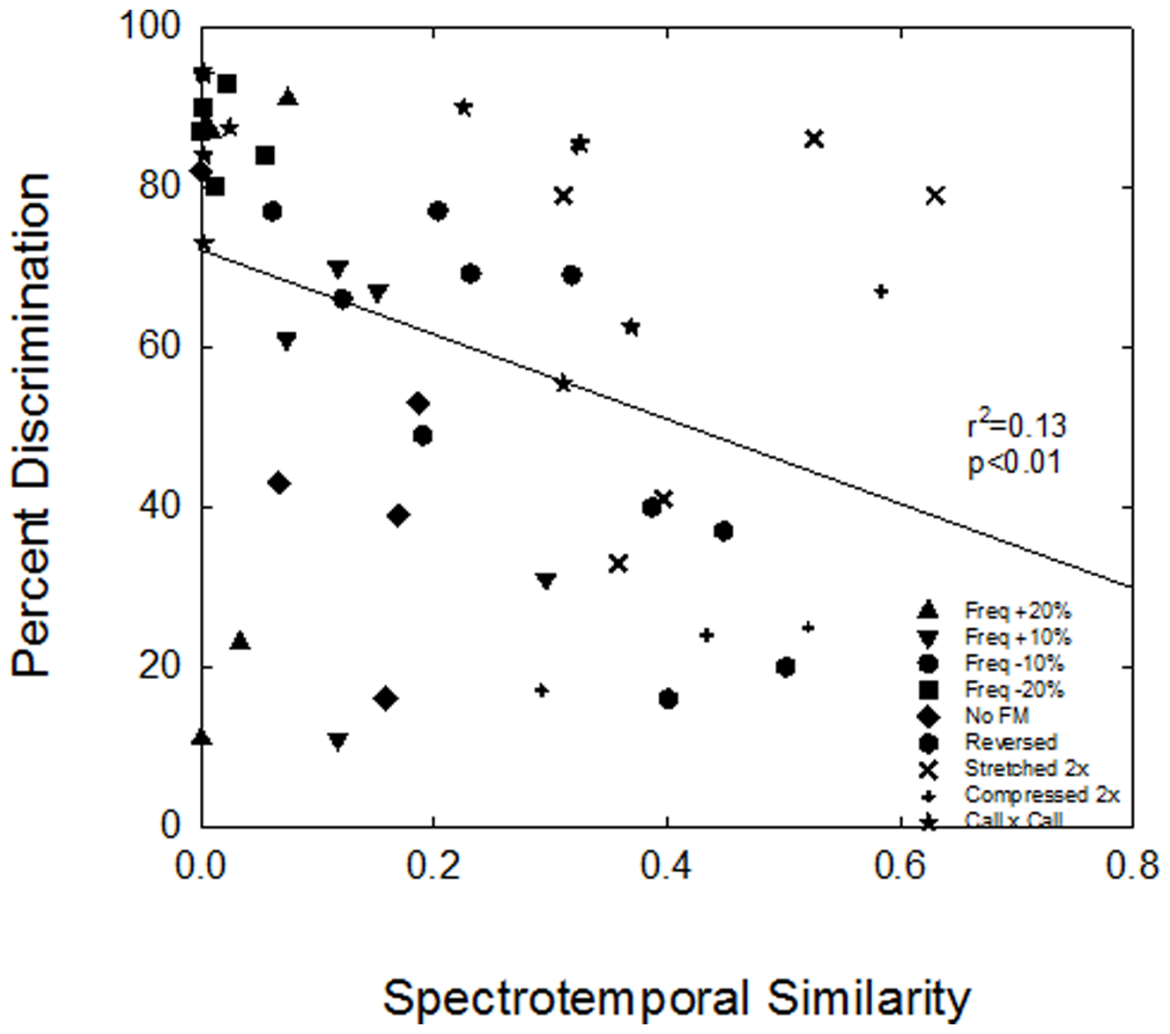
Correlation between spectrotemporal similarity and discrimination performance. Percent correct discrimination as a function of spectrotemporal similarity for all vocalization discriminations in the first experiment and all vocalizations versus their manipulations in the second experiment.

Spectrotemporal similarity between the different vocalizations and between the vocalizations and their manipulations were significantly correlated with discrimination performance (Figure 5, *r^2^*  =  0.13, *p*<0.01). For example, discrimination performance was >80% for the vocalizations that were most dissimilar. In general, as spectrotemporal similarity between vocalizations increased, discrimination performance decreased. Thus, the mice have the behavioral ability to discriminate between vocalizations that are spectrotemporally dissimilar from one another, and this ability declines for more similar vocalizations.

## Discussion

We tested the ability of mice to discriminate between regularly emitted USVs using operant conditioning procedures and the psychophysical Method of Constant Stimuli. We found that mice can reliably discriminate between vocalizations, particularly those with dissimilar spectrotemporal properties. Thus, although the functional importance of different types of mouse vocalizations is not yet known, our findings show that mice are able to behaviorally discriminate vocalizations, suggesting that the different types of vocalizations are perceptually meaningful.

Until now, the only discrimination studies using mouse USVs have been conducted using preference studies. These studies have shown that female mice spend less time with a devocalized male than one that is vocalizing [14], females pair-housed with males later preferred cage areas projecting male vocalizations [15], female Neotropical mice (genus *Scotinomys*) prefer signals produced at a fast rate over a slow rate [16], female mice prefer synthetic multiharmonic calls that are similar to natural pup calls [17], female mice respond to ultrasonic ‘songs’ with approach behavior [18] and finally, female wild house mice can distinguish between ultrasonic vocalizations produced by their brothers and those from unfamiliar non-kin [19]. However, these types of preference experiments are limited in their ability to tell us about acoustic communication. These studies show large variations in results across laboratories, animals can only be tested once or twice and only for a minute or two before they habituate, and actual amounts of time spent in one compartment compared to another are often quite small (reviewed in [15]). Further, when an animal spends more time in proximity of one stream of vocalizations over another, we know nothing about why it is doing this. Similarly, the finding of no preferences may be because the animal cannot discriminate between two stimuli. For these reasons, psychophysical experiments using reliable observers are much more informative for understanding rodent communication.

It is well known that mice emit ultrasonic vocalizations in a variety of social contexts (reviewed in [3]), and that they emit a number of different vocalization types that have been referred to as syllables [20]. The classification of syllables has been based on spectrotemporal properties of the vocalizations [4], [5], [7] or statistical clustering algorithms [6], [21], rather than on the ability of mice to behaviorally discriminate between different vocalizations. To the best of our knowledge, this is the first study to show that mice can behaviorally discriminate among a small number of commonly emitted vocalizations. This has been an open question since Ehret's early studies [22] on how lactating female mice do not behaviorally differentiate between narrowband noise models and natural calls in a phonotaxis task. Until now, it was uncertain if mice were able to discriminate between these artificial models and natural calls. The current study suggests that if sufficiently motivated, mice can in fact make subtle acoustic discriminations.

In general, the mice were best able to discriminate between vocalizations that had dissimilar spectrotemporal properties. The mice were able to accurately discriminate between the upsweep vocalizations and the harmonic and jump vocalizations, but were not able to accurately discriminate between the two upsweep vocalizations or among the harmonic and jump vocalizations. This is illustrated by the multidimensional map of perceptual space for the five vocalizations. The upsweep vocalizations occupied the negative space while the harmonic vocalizations occupied the positive space along dimension one. This analysis also suggests that peak frequency was of less importance for discrimination behavior than the presence or absence of jumps in the harmonic vocalizations, as the two harmonic vocalizations with jumps were perceived as more similar to each other than they were to the harmonic vocalization without jumps. However, frequency does have some relevance, as the two 30 kHz vocalizations occupy positive space in dimension 2, while the 40 kHz harmonic vocalization occupies negative space in the same dimension. These multidimensional scaling results from mice are similar to those from similar studies conducted in budgerigars (*Melopsittacus undulatus*, [13]), where different vocalization “types” occupy different perceptual space, simply measured by discrimination performance. Thus, this type of study provides access to the animals’ perceptual capabilities, information not obtained by other experimental methodologies.

Our behavioral discrimination results match well with neural discrimination results obtained in the IC of CBA/CaJ mice [9]. Because the same vocalization stimuli were used in the behavioral and neurophysiological studies, we can gain a better understanding of the neural correlates of behavioral discrimination of vocalizations in mice. Our finding that mice could accurately discriminate between the upsweeps and the harmonic vocalizations corresponds well to the findings that more IC neurons fire to the harmonic vocalizations compared to the upsweeps and that there are differences in the reliability of the temporal firing patterns between the stimuli. Thus, IC neurons can discriminate between the upsweep and harmonic vocalizations, just like the awake behaving mice can. While the mice were less accurate at discriminating between the two upsweep vocalizations, their performance was greater than chance (56% versus 10%), indicating some level of discrimination ability. This result matches somewhat with the finding that more IC neurons responded to the female-emitted upsweep vocalization than to the male-emitted upsweep vocalization [9]. Behavioral performance may have been less accurate than the neural performance because the upsweeps were both short in duration and very high frequency, and it is well known that behavioral discrimination is better with longer duration stimuli (e.g. [23]) and there is little neural representation of vocalizations above 60 kHz in the mouse auditory system [24].

To better understand what spectrotemporal properties in vocalizations are important for behavioral discrimination ability, we independently manipulated parameters within each of the vocalization stimuli and tested how well the mice could discriminate the original vocalization from the modified one. The mice were able to discriminate all of the manipulations with accuracy above chance, although the mice were better able to discriminate between the original and modified vocalization when frequency content was altered. These behavioral results are very similar to how neurons in the IC responded to the same original and modified vocalizations we used in this study [9]. Neurons changed their discharge rate, discharge pattern, or both when the vocalizations were shifted in frequency, when frequency modulation was removed, or when duration was altered. In general, shifting frequency content led to the greatest change in neural response patterns.

Overall, behavioral performance for the five vocalizations and their spectrotemporal manipulations shows that vocalization shape, frequency content, and the presence or absence of frequency jumps all affect the mouse’s ability to discriminate among different vocalizations. Discrimination performance was lower for spectrotemporally similar vocalizations and higher for spectrotemporally dissimilar vocalizations. Thus, our results provide evidence that mice are capable of perceptually discriminating among commonly emitted vocalizations that differ in spectrotemporal properties. Furthermore, our behavioral results closely match neural responses in the IC, suggesting that the neural mechanisms underlying selectivity to vocalizations in the IC play a fundamental role in the perception of vocalizations.
